# Kawasaki disease and the environment: an enigmatic interplay

**DOI:** 10.3389/fimmu.2023.1259094

**Published:** 2023-12-18

**Authors:** Ridhima Aggarwal, Rakesh Kumar Pilania, Saniya Sharma, Amit Kumar, Manpreet Dhaliwal, Amit Rawat, Surjit Singh

**Affiliations:** Allergy Immunology Unit, Department of Pediatrics, Advanced Pediatrics Centre, Post Graduate Institute of Medical Education and Research, Chandigarh, India

**Keywords:** Kawasaki disease, environment, genetics, male, season

## Abstract

Kawasaki disease (KD) is a common systemic vasculitis of childhood. Although it has been almost 6 decades since Dr. Tomisaku Kawasaki reported the first case series of KD, the underlying cause remains a mystery. KD is a self-limiting disease. However, a dreaded complication is development of coronary artery abnormalities (CAAs). KD is the most common cause of acquired heart disease in children in the developed world and is being increasingly reported from developing countries too. Over the years, significant observations have been made about epidemiology of KD. It usually affects children below 5, has male preponderance and has significantly higher incidence in North East Asian countries. While several hypotheses have been proffered for etiology of KD, none have been conclusive. These include associations of KD epidemics in Japan and the United Stated with changes in tropospheric wind patterns suggesting wind-borne agents, global studies showing peaks of incidence related to season, and increased rates in populations with a higher socioeconomic profile related to hygiene hypothesis and vaccination. Furthermore, the self-limiting, febrile nature of KD suggests an infectious etiology, more so with sudden decline noted in cases in Japan with onset of COVID-19 mitigation measures. Finally, single nucleotide polymorphisms have been identified as possible risk alleles in patients with KD and their significance in the pathogenesis of this disease are also being defined. The purpose of this review is to elucidate the puzzling associations of KD with different environmental factors. Looking at patterns associated with KD may help us better predict and understand this disease.

## Introduction

**Figure 1:**
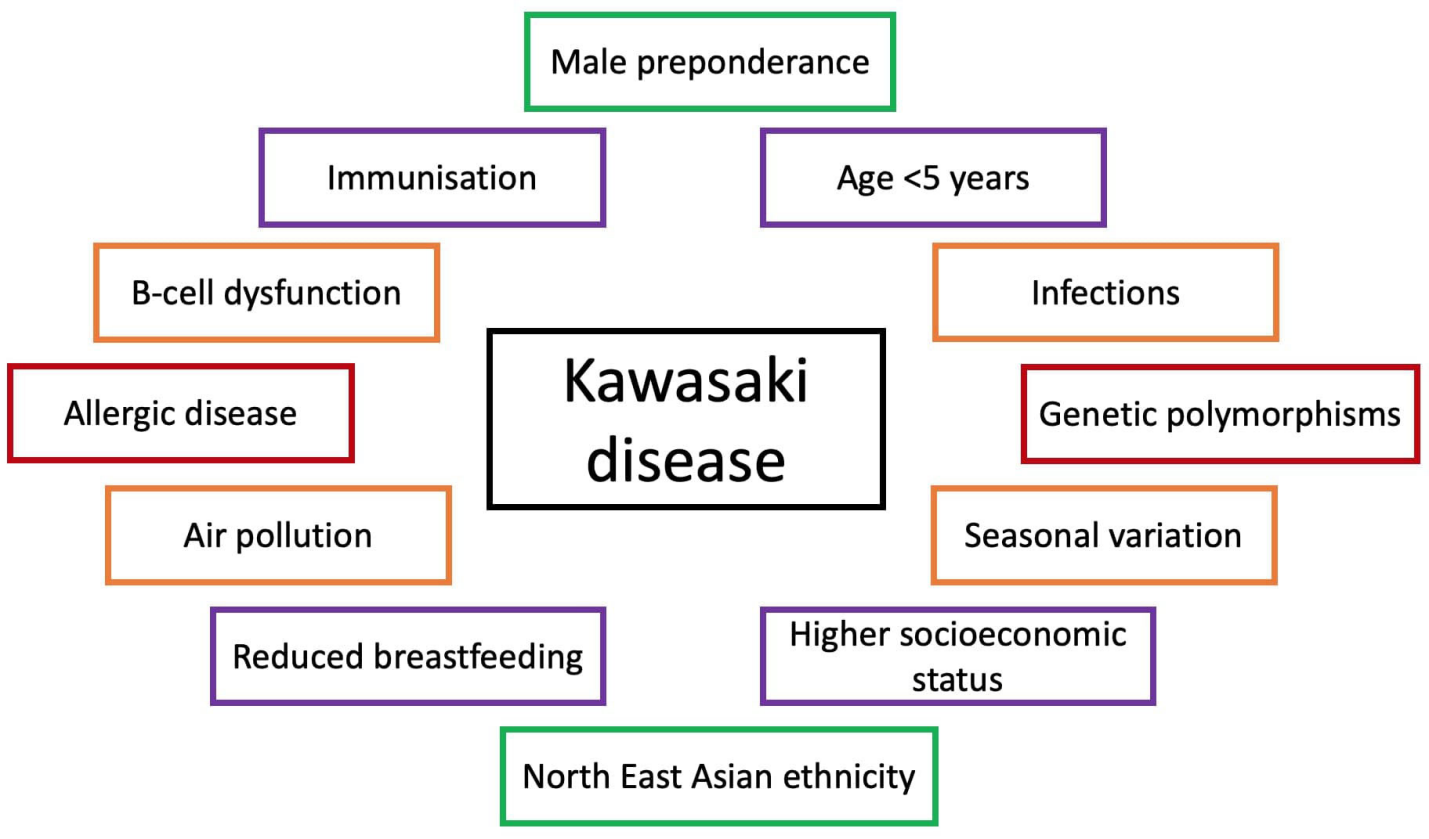
Factors implicated in pathogenesis of Kawasaki disease.

Kawasaki disease (KD) was first described by Dr. Tomisaku Kawasaki in 1967 as “an acute febrile mucocutaneous lymph node syndrome with unique digital desquamation” and has remained an enigma ever since (1). More than half a century later, with advances in technology and increased awareness, physicians are better able to recognize and treat this syndrome (2, 3). However, the underlying cause still remains unknown. KD is a pediatric, self-limited systemic vasculitis with several dreaded complications such as the development of coronary artery abnormalities (CAAs) and myocarditis (4). It stands to be the most common cause of acquired heart disease in the developed world, with its unique predilection for involvement of coronary arteries (5). Over the years, several observations on the interplay between KD and the environment have been made, leading to a widely accepted hypothesis that it occurs as an exaggerated inflammatory response to certain environmental triggers in genetically susceptible individuals. The aim of this manuscript is to review various associations of KD with environmental factors (**Figure 1**). This may help to understand and predict situations that lead up to the occurrence of this disease.

## Age and gender distribution

KD usually affects children, with a distinct predilection for boys and almost 85% of cases occurring in children below 5. It predominantly presents as a monophasic, self-limiting febrile illness (6). Age and gender play an important role as risk factors for complications, as age less than 1 year or above 9 years, and male gender have universally been identified to be significant risk factors for development of CAAs (7–12). KD has been postulated to occur due to ‘dysregulation of early B cell development’ as KD susceptibility genes identified by genome wide association studies (GWAS) are involved in B cell development (13). *FCGR2A* is one such gene, which encodes low-affinity Fc gamma receptor (FcγRIIa) that binds to Fc portion of IgG. It is usually present on macrophages and neutrophils, and involved in clearance of circulating immune complexes. However, it is also involved in transducing intracellular signals via its’ immunoreceptor tyrosine-based activation motifs (ITAM) in cytoplasmic domains in B-cells. Co-ligation of B-cell receptor and FcγRIIa stops further B cell activation. Furthermore, soluble FcγRIIa inhibits B-cell proliferation and immunoglobulin production. The “A” allele of *FCGR2A* single nucleotide polymorphism (SNP - rs1801274), reportedly binds with high-affinity and incidentally also has a significant association with KD, particularly in boys below 1 year of age (14). This may explain the skewed incidence of KD in male infants.

## Role of infectious triggers and its implications

Most widely accepted theory in pathogenesis of KD is that it occurs due to an unidentified ubiquitous organism that affects genetically predisposed individuals. The clinical observation that it presents as sudden onset high-grade fever in otherwise healthy children, with rarity of recurrence and self-limiting disease course is highly suggestive of an infective trigger (15). Various infectious triggers such as parainfluenza, varicella and superantigens of staphylococci and streptococci have been previously identified (16–20). Epidemiological observations that KD has had major epidemics and has temporal and spatial clustering is characteristic for an infection being spread by respiratory route (15).

Robust evidence for an unidentified infectious agent, entering via respiratory route and showing tropism for vascular tissues was provided by Rowley et al. when they discovered significantly increased IgA-specific plasma cells, CD8 T-lymphocytes and macrophages infiltrating the coronary arteries and upper respiratory tracts of patients with KD in acute phase in comparison to controls (21). Further investigators sequenced IgA genes from plasma cells from arteries of patients with acute fatal KD and reported an oligoclonal IgA response (11). Subsequently, these oligoclonal antibodies were synthesized *in vitro*, and used to perform immunohistochemistry experiments on bronchial tissues from patients with KD in acute phase and control patients. Specific binding of these antibodies to cytoplasmic antigens in patients with KD was noted in comparison to controls (22). Further, inclusion bodies were found in ciliated bronchial epithelium in patients with acute KD, and when tested with the specific synthetic IgA antibody found that the antigen was localised to these cytoplasmic inclusion bodies itself. This was considered consistent with aggregates of viral proteins or nucleic acid which may be responsible for triggering KD (23). This gave rise to an infection based model of KD pathogenesis, wherein an ubiquitous agent infects the ciliated bronchial epithelium, forming inclusion bodies, causing disease in genetically susceptible individual by entering blood stream and targeting blood vessels (24). This model explained acquired immunity as an explanation for the lack of recurrence and shedding of viral agent and community contact as cause for epidemics.

Epidemiological evidence corroborating this theory came with onset of the COVID-19 pandemic when it was found that although annual numbers of patients with KD in Japan had increased consistently from 2001 – 2019, there was an abrupt decrease in number of cases and incidence rates in 2020 (25). Investigators ruled out factors such as decreased hospital visits due to parental hesitancy during the pandemic and still found 35.6% reduction in KD between 2019 and 2020, which was indeed significant. Monthly analysis of year 2020 revealed number of KD patients markedly reduced during COVID-19 special mitigation measures period, especially for children aged >24 months and rebounded much faster for the same age group at end of special-mitigation measures (25). This highlights that mask-wearing and nation-wide closure of schools may have prevented an infectious trigger of KD in these children. This could also mean infection control measures, such as mask-wearing, hand washing and social distancing could help prevent development of KD among older children (25).

## Temporal and spatial clustering cluster

There is evidence to support aggregation of cases of KD in both time and space, further strengthening the theory that KD may have an infectious origin. Clustering of cases over 14 years was studied in Japanese children, and obvious patterns were observed, including seasonality with peaks in January and July that remained consistent in observation (26). Temporal clustering was evident with nationwide outbreaks, including 3 major epidemics reported in Japan (26). Furthermore, spatial clustering was noted in years 2007-2010 and 2012 in Tokyo metropolitan area (27). Similarly, Rypdal et al. demonstrated significant grouping of cases within the time-space interval of 10 days and 10 – 100 kilometres area in patients with KD from California over a period of 15 years (28). Furthermore, clustering of development of CAAs as a complication of KD has also been observed in cities from Canada at distinctly different time periods (29). Epidemiological examination of the distribution of KD across time and space is significant, and may help uncover the etiology or pathogenesis of this condition.

## Ethnicity and genetics as predisposing factors

Japan has the highest annual incidence of 371 per 100,000 children aged 0-5 years (25), followed by Korea (194.7 per 100,000 children aged 0-5 years) (30) and Taiwan (69.0 per 100,000 children aged 0-5 years) (31). The annual incidence in Japan is 10 times higher than that in the US (20.8 per 100,000 children aged 0-5 years) (32). These data suggest that KD has a strong correlation with Asian ethnicity – whether this is related to susceptible genes in this population or due to exposure to specific agents in lifestyle common in the North East Asian population is yet to be elucidated (33). However, the strongest evidence for ethnicity and possibly genetics playing a role came from epidemiological data reported from Hawaii when it was noted that the annual incidence of KD amongst Japanese Americans in Hawaii from 1996-2006 was 210.5 per 100,000 children below 5, while the incidence amongst Caucasian children in the same period in Hawaii was only 13.7 per 100,000 children below 5 (34). Higher incidence of KD in Asian population even in the transmigration area (having an almost similar incidence to that seen in mainland Japan) was suggestive that difference in incidence among different ethnicities stems from genetic factors rather than just from the environment. In addition, familial aggregation of KD points towards genetic susceptibility. When compared to general population, siblings of a KD patient have a 10-30 times higher chance of developing KD (35), and the proportion of having a past history of KD was twice as high in parents of a KD patient than in general population (36).

The role of increased T-cell activation through the Ca^2+/^NFAT pathway in determining susceptibility to KD was suspected when functional SNPs in the inositol 1,4,5-trisphosphate 3-kinase C (ITPKC) gene (37) and the caspase-3 (CASP3) gene (38) were identified to be significantly associated with incidence as well as increased risk of coronary artery lesions in children with KD. Subsequently, high-risk genetic polymorphisms in the *ITKPC* gene associated with KD were demonstrated to cause higher stimulated intracellular calcium levels and increased production of IL-1b and IL-18 (39). This explained the efficacy of IL-1 blockade in recalcitrant KD. Onouchi et al. carried out GWAS on KD patients from Japan and identified 6 susceptibility genes; *FCGR2A, CASP3, HLA, BLK, ITPKC, CD40* (40). It was noted that some of these genes were related to early development and function of B-cells. This suggested that B cell immunity could also be crucial for development of KD, and that intravenous immunoglobulin (IVIg) would be required for adequate treatment of the same (13). However, contrary to expectations, when risk allele frequencies of the susceptibility SNPs for these 6 genes was studied in Taiwanese and European population, frequencies were actually comparable, and in some instances even higher than those in Asians (33). These findings hint that KD is not completely governed by genetics, and there is likely an environmental trigger also affecting predisposed individuals.

## Socioeconomic status and the hygiene hypothesis

Through the years observations regarding socioeconomic status of patients affected with KD have been reported. The first report in 1989 was on 106 patients of KD by Ichida et al. from New York. Authors noted that there were more families of children with KD that were members of upper and middle class (73%) than families of the population seen in general pediatrics (31.7%) in their hospital (41). Subsequently, Prakash et al. compared variables between families with KD and other rheumatological illnesses and found male sex, higher education status of parents, urban residence, complete immunisation and higher socio-economic status to be notably associated with KD (42). Fujiwara et al. reported similar findings from a cohort in Japan wherein they found children born in households with higher income, smaller family size and urbanization at birth were significantly associated with an increase in incidence of KD (43).

Families with higher income, higher education status of parents, smaller family size and urban residence are likely linked to less overcrowding, high-quality housing and decreased risk for early pediatric infections. The hygiene hypothesis suggests that along with a sterile living environment, excessive use of antibiotics, formula feeding and reduced early exposed to infectious agents results in a defective B cell development (13). Lack of a well-developed B cell immunity increases susceptibility to environmental triggers that are non-pathogenic in healthy individuals become triggers for development of KD in these children (13). This theory is further supported as susceptibility SNPs in genes identified on GWAS in Japanese population were all in genes involved in early B-cell development (33). Furthermore, corroborating evidence lies in the peak incidence of KD being seen at the age of lowest immunoglobulin levels (9-12 months), protective efficacy of vaccination and breastfeeding and efficacy of treatment with IVIg. Moreover, there is published literature on allergic diseases being more common in patients with a past history of KD (44–47). This is likely due to absence of early-life exposure to microbes that leads to increased Th2 skewing and a higher incidence allergic disease. Association of KD and allergic disease again is likely due to common early-life and socioeconomic determinants (48).

## Breastfeeding

Studies have reported a protective effect of breastfeeding for 6 months or more on occurrence of KD. Breastfeeding has also been associated with shorter duration of fever, higher pre-IVIg albumin levels and a reduced risk of developing CAAs (49–51). This may simply be attributed to overall anti-inflammatory effect of breast milk and breastfeeding. However, it also helps in formation of probiotic bacteria in the infantile gut, especially Lactobacillus. The SCFAs abundantly present in breastmilk also help in activation of Treg cells. Overall, it also stimulates a strong and mature Th1 response, and reduces the inherent intrauterine Th2 predominant response to create a more balanced and regulated adaptive immunity.

## Immunisation

Studying the relation between KD and immunisation has been challenging, due to lack of a standard case definition for reporting KD as an adverse reaction to vaccination. Thus, temporal association between KD and vaccination may have been reported in the past but causal association has never been accepted. Even though a greater than expected number of ‘possible KD’ cases have been counted in various adverse event following vaccination reporting systems, an increased risk of KD following vaccination is undocumented. This is because the incidence of KD after vaccination did not differ significantly from the existing background population incidence in these studies (52–54).

## Gut microbiota

The colonisation of essential symbiotic intestinal microbiota depends entirely on expected post-natal exposure through vaginal delivery, maternal gut microbiota and breastfeeding. These colonies are easily affected and changed by practices such as formula feeding, excessive antibiotic use and reduced environmental exposure (55). The critical period for achieving this symbiosis is in first 1000 days of life (56), that incidentally also coincides with the age group most affected by KD. Furthermore, environmental factors such as higher per capita income, small family size and urbanisation are associated with increased dysbiosis of the gut and have already been associated with a risk of KD as well (43).

Abnormally increased gram-positive cocci (Streptococcus and Staphylococcus) and reduced Lactobacilli have been demonstrated in children with KD in comparison to febrile and healthy controls (57–59). SCFAs produced by gut microbiota regulate Th17 and Treg differentiation. Loss of balanced symbiotic status with gut microbiota would lead to a reduced production of SCFAs and is hypothesized to cause an aberrant immune response, possibly involved in pathogenesis of KD (60). These studies suggest that a disruption in the balance of the gut microbiota may hinder innate immunity and development of a robust adaptive immunity, that may selectively drive the development of KD in certain children.

## Airborne agents

In 2011, Rodo et al. analysed 3 dramatic KD epidemics in Japan in April 1979, May 1982, and March 1986 and found that prior to the beginning of epidemics the wind patterns corresponded to the typical summer climatological configuration – i.e. from south, blowing across Japan. However, after beginning of autumn, specifically and only in these 3 years, there was a major shift in the wind direction and the winds turned in north-west direction. It was in these 3 years, major epidemics in Japan were reported with a sharp increase and deviation from the gradually rising incidence rates of KD in the preceding years. Again, as the winds shifted and blew from south in following years, a marked decrease in KD incidence was noted. This resulted in a return to the epidemiological pattern that had been existing before 3 nationwide epidemics (61). Similarly, Jorquera et al. also reported a statistical association of KD at Santiago, Chile with tropospheric, northerly wind patterns (62). Furthermore, Rodo et al. analysed tropospheric aerosols in comparison to ground aerosols by conducting sampling campaigns during KD seasons over Japan, and not only reported major differences between the two, but also a high percentage of Candida (i.e. 54% of all fungal species) in tropospheric samples (63). Following this, Candida sp. derived substances were demonstrated to induce coronary vasculitis in experimental mice models (64, 65). This implies that the putative causative agent, possibly a fungal toxin, is transported across by strong air currents that develop in upper troposphere, supporting the theory that KD may be caused by an ubiquitous air-borne infectious organism.

In contrast, several studies have also documented positive associations between daily variations in ambient air pollution and KD. Increased concentrations of pollutants such as sulphur dioxide and nitric oxide, higher temperatures, and increased concentrations of particulate matter 2.5μm in diameter have been reported to be associated with an increased incidence in KD (66–68). In addition, Alphonse et al. suggested the role of mercury as an air borne pollutant associated with increased prevalence of KD and also demonstrated its role in causing increased incidence and severity of coronary artery lesions (69). Thus, the etiological agent of KD may surely be windborne, but whether it is a virus, fungus, inert particle, pollutant or pollen is yet to be determined.

## Seasons

Yanagawa et al. first noted seasonal peaks in Japan that began in December and settled in March (70). Since then, although seasonal variation has been extensively studied in Japan (26), the cause for this persistent pattern remains a mystery. In addition, it has been reported that incidence of KD in San Diego also follows a seasonal cycle similar to that seen in Japan, with main peak occurring between January to March and lowest point in cases occurring in September and October (28). In a major epidemiological study by Burns et al, global mapping of seasonal peaks and troughs of KD incidence was done (71). The study group found that aggregated monthly means from Northern hemisphere showed highest incidence in January through March which was around 40% higher than in months of lowest incidence (August through October) with high statistical significance. Even though their data sets from the tropics and Southern hemisphere were not entirely convincing, they found evidence that the incidence was maximum in May through June – with an approximately 30% higher incidence than months of lowest incidence (February, March and October). Seasonality of KD was also studied by Singh et al, where incidence of KD reported to be was highest in the months of October and November in Chandigarh, India consistently over two decades, even as awareness and rate of diagnosis increased (72). Consistency in KD seasonality across hemispheres is suggestive of an environmental agent that maybe causative of this disease across distant regions and ethnicities and points to a process that operates over a hemispheric scale.

## Summary

KD is a vasculitis that occurs more often in children below 5 with higher incidence noted in males and children of Asian ethnicity. While breastfeeding has been identified to have a protective role, lack of exposure to antigens, increased sanitation, gut dysbiosis and higher socio-economic status were associated with a higher incidence of this disease. Published literature suggests KD most likely occurs due to an unidentified, windborne agent in those that are genetically susceptible. Overall, KD is a systemic vasculitis of childhood with no identified etiological agent yet. However, multiple enigmatic associations have been described that help us understand and predict the disease course and severity better.

## Author contributions

RA: Writing – original draft. RP: Writing – original draft, Conceptualization, Supervision, Validation, Writing – review & editing. SSh: Writing – review & editing. AK: Writing – review & editing. MD: Writing – review & editing. AR: Writing – review & editing. SSi: Supervision, Writing – review & editing.
